# Isolation of biologically active constituents from *Moringa peregrina* (Forssk.) Fiori. (family: Moringaceae) growing in Egypt

**DOI:** 10.4103/0973-1296.80667

**Published:** 2011

**Authors:** Taha S. El-Alfy, Shahira M. Ezzat, Ahmed K. Hegazy, Aziza M. M. Amer, Gehan M. Kamel

**Affiliations:** *Department of Pharmacognosy, Faculty of Pharmacy, Cairo University, Kasr-El-Ainy, Cairo - 11562, Egypt*; 1 *Department of Botany, Faculty of Science, Cairo University, Giza - 1221, Egypt*; 2 *Department of Pharmacology, Faculty of Vetrenary Medicine, Cairo University, Giza - 1221, Egypt*

**Keywords:** Anticancer, antihyperglycemic, *Moringa peregrina*, rhamnetin-3-O-rutinoside, 6-methoxy-acacetin-8-C-β-glucoside

## Abstract

**Background:**

*Moringa peregrina* is a wild plant that grown in the eastern desert mountains in Egypt. Although, this plant is native to Egypt, no details studies were traced on its chemical composition and biological activity.

**Materials and Methods:**

The different fractions of the ethanolic extract of the dried aerial parts of the plants were subjected to fractionation and purification on various silica and sephadex columns for the isolation of the major compounds which were tested for there anticancer activity. The aqueous and ethanolic extract as well as its different fractions were tested for antihyperglycemic effect on Streptozitocin-induced diabetes in rats.

**Results:**

Investigation of the different fractions of the ethanolic extract of the aerial parts of *M. peregrina* yielded lupeol acetate (1), β-amyrin (2), α-amyrin (3), β-sitosterol (4), β-sitosterol-3-O-glucoside (5), apigenin (6), rhamnetin (7), neochlorogenic acid (10), rhamnetin-3-O-rutinoside (12), and 6-methoxy-acacetin-8-C-β-glucoside (13) which were isolated for the first time from the plant. Compound (13) was isolated for the first time from genus *Moringa*. In addition, quercetin (8), chryseriol-7-O-rhamnoside (9) and quercetin-3-O-rutinoside (11) were also isolated. Identification has been established by spectral data (UV, MS, IR, 1H, 1H -1H COSY, and 13C-NMR). The major isolated compounds were found to have valuable cytotoxic activities against breast (MCF 7) and colon (HCT 116) cancer cell lines and their activities were comparable to the reference drug doxorubicin. On the other hand, the aqueous and ethanolic extracts as well as the n-hexane fraction were found to have potent antihyperglycemic effect on Streptozitocin-induced diabetes in rats.

**Conclusion:**

The Egyptian plant *M. peregrina* is rich in biologically active ingredients which showed potent cytotoxic activity and also its ethanolic extraxt exert a significant antihyperglycemic effect.

## INTRODUCTION

On reviewing the literature of *Moringa peregrina* (Forssk.) Fiori, a single report was found about the antihyperglycemic effect of the ethanolic extract of the defatted aerial parts from which quercetin, quercetin-3-O-rutinoside, chryseriol-7-O-rhamnoside, and 6, 8, 3’,5’-tetramethoxy apigenin were isolated.[1] In this article, the separation and characterization of 10 compounds isolated for the first time from *M. peregrina* is reported, in addition to 3 compounds that were isolated before from the same plant. Also, the acute toxicity and antihyperglycemic effect of the aqueous extract, ethanolic extracts, and its fractions, as well as the cytotoxic effect of the different fractions and the major isolated compounds are presented.

## MATERIALS AND METHODS

UV spectra were measured using a Shimadzu UV 240 (P/N 204-58000) spectrophotometer (USA). Mass spectra were measured using Shimadzu QP-2010 Plus (USA). NMR spectra were recorded at 300 (^1^H) and 75 MHz (^13^C) on a Varian Mercury-300 instrument (Switzerland). The NMR spectra were recorded in CDCl_3_ or DMSO-*d*_6_, and chemical shifts were given in δ (ppm) relative to TMS (Trimethylsulphoxide) as internal standard. Electrothermal 9100 (United Kingdom) was used for the determination of melting points (mp) (uncorrected).

Authentic sterols and triterpenes were obtained from E. Merck (Darmstadt, Germany). Silica gel H (E-Merck, Darmstadt, Germany) for vacuum liquid chromatography (VLC) and silica gel 60 (Fluka, 70-230 mesh ASTM, Germany) and Sephadex LH-20 (Pharmacia, Uppsala, Sweden) for column chromatography were used. Thin-layer chromatography (TLC) was performed on silica gel GF_254_ precoated plates (Fluka, Germany). The chromatograms were visualized under UV light (at 254 and 366 nm) before and after exposure to ammonia vapor, as well as spraying with anisaldehyde-sulfuric acid spray reagent.

### Plant material

The aerial parts of *M. peregrina* (Forssk.) Fiori (Family: *Moringa*ceae) were collected in the spring from the eastern desert mountains, Egypt. The plant was identified by Prof. Dr. Ahmed Hegazy, Head of the Department of Botany, Faculty of Science, University of Cairo, Egypt. The collected material was air-dried, reduced to powder and kept for extraction.

### Extraction and isolation

The air-dried aerial parts (650 g) were powdered and then extracted by percolation with 95% ethanol (4 × 7 L) to yield (250 g) ethanolic extract residue. The residue (200 g) was suspended in distilled water and partitioned between *n*-hexane, chloroform, ethyl acetate, and *n*-butanol (saturated with water). The solvents were separately evaporated under reduced pressure to yield 6, 3, 4.7, and 5 g, respectively.

**n-Hexane fraction (HF):** Four grams was chromatographed over a VLC (Si gel H, 30 g, 5 × 3 cm). Gradient elution was carried out using n-hexane-chloroform mixtures and chloroform-ethyl acetate mixtures. Fractions 100 mL each were collected to yield 4 main fractions (A-D). **Fraction A** (15%-20% chloroform-n-hexane, 0.5 g) was rechromatographed over a Si gel 60 column (25 × 2 cm, 50 g), using n-hexane as an eluent to give compound 1 (18 mg). **Fraction B** (25%-30% chloroform-n-hexane, 0.6 g) was rechromatographed over a Si gel 60 column (25 × 2 cm, 50 g), using n-hexane-ethyl acetate (9.9:0.1 v/v) as an eluent to give compound 2 (15 mg). **Fraction C** (40% chloroform-n-hexane, 1.2 g) was purified by passing several times over Sephadex LH-20 columns (40 × 2 cm) using chloroform-methanol (1:1 v/v) as an eluent. The purified fraction was rechromatographed over a Si gel 60 column (25 × 2 cm, 50 g), using n-hexane-ethyl acetate (9.5:0.5 v/v) as an eluent to yield compound **3** (20 mg) and compound 4 (33 mg). **Fraction D** (100% ethyl acetate, 0.9 g) was rechromatographed over a Si gel 60 column (25 × 2 cm, 50 g), using chloroform-methanol (9.6:0.4 v/v) as an eluent to give white powder of compound **5** (35 mg). **Chloroform fraction (CF):** Two grams was chromatographed over VLC column as mentioned under the n-hexane extract to yield compounds **4** and **5**. **Ethyl acetate fraction (EF):** Two grams was fractionated over a Sephadex LH-20 column (25 × 3 cm) using 20%, 40%, 60%, and 80% methanol in water mixtures as an eluent. Fractions (200 mL) were collected to yield 3 main fractions **(E-G)**. These fractions were purified by passing several times over Sephadex LH-20 columns, using methanol as an eluent to yield compounds **6** (24 mg), **7** (17 mg), **8** (15 mg), and **9** (12 mg). ***n*-Butanol fraction (BF):** Four grams was fractionated over a Sephadex LH-20 column as under the ethyl acetate fraction to yield 3 main fractions (H-J). These fractions were purified by passing several times over Sephadex LH-20 columns, using methanol and methanol-water mixtures (1:1 v/v) as an eluent to yield compounds **10** (28 mg), **11** (10 mg), **12** (45 mg), and **13** (30 mg), respectively.

**Compound 1**

White microcrystalline powder.

mp: 222-224°C.

Rf: 0.51(n-hexane-ethyl acetate 9.5:0.5).

IR (KBr): 3400, 3240, 1725, 1689.

MS (EI, 70 eV): *m/z* (%) = 468.2 [M]^+^ (7.9), 408 [M-CH_3_ COO]^+^ (40), 218 (56), 203 (77), 189 (100).

**Compound 2**

White needle crystals (n-hexane).

mp: 195-197°C.

Rf: 0.56 (n-hexane-ethyl acetate 9:1).

IR (KBr): 3400, 3242, 1690.

MS (EI, 70 eV): *m/z* (%) = 426.1 [M]^+^ (10), 218 (100), 203 (79), and 189 (60).

**Compound 3**

White needle crystals (n-hexane).

mp:185-186°C.

Rf: 0.4 (n-hexane-ethyl acetate 9:1).

IR (KBr): 3400, 3242, 1690.

MS (EI, 70 eV): *m/z* (%) = 426 [M]^+^ (12.3), 218 (100), 203 (38.46), and 189 (34.61).

**Compound 4**

White needle crystals (n-hexane).

mp: 140-141°C.

Rf: 0.25 (n-hexane-ethyl acetate 9:1).

IR (KBr): 3400, 3242, 1690, 1212, 1051, 1022, 953.

MS (EI, 70 eV): *m/z* (%) = 414 [M]^+^ (100), 396 (51), 329 (42), 303 (44), 273 (60), and 255 (80).

**Compound 5**

White microcrystalline powder.

mp: 290°C.

Rf: 0.37 (chloroform-methanol 9.5:0.5).

IR (KBr): 3400, 3242, 1690, 1212, 1051, 1022, 953.

^1^H-NMR (300 MHz, DMSO): 0.66 (3H, d, *J* = 5.5 Hz, Me-21), 0.78 (3H, t, *J* = 6.3 Hz, Me-29), 0.83 (3H, d, *J* = 6.2 Hz, Me-26), 0.90 (3H, d, *J* = 6.3 Hz, Me-27), 0.92 (3H, s, Me-18), 0.96 (3H, s, Me-19), 3.03 (1H, m, H-3), 4.21 (1H, d, *J* = 7.5, H-1’), 5.33 (H, br s, H-6) ppm.

**Compound 6**

Yellow microcrystalline powder.

mp: 348-350°C.

Rf: 0.45 (chloroform-methanol 9.5:0.5).

IR (KBr): 3300, 3050, 2920, 1660, 1620, 1510, 1360, 1060, 910.

UV-Vis λ_max_ nm: (MeOH) 266, 296sh, 336 (NaOMe) 274, 324, 390 (AlCl_3_) 274, 299, 386 (AlCl_3_ /HCl) 274, 299, 382 (NaOAc) 271, 300, 376 (NaOAc-H_3_ BO_3_) 269, 301sh, 338.

**Compound 7**

Yellow microcrystalline powder.

mp: 294-296°C.

Rf: 0.42 (chloroform-methanol 9.5:0.5).

IR (KBr): 3295, 3050, 2920, 1640, 1620, 1510, 1360, 1060, 910.

UV-Vis λ_max_ nm: (MeOH) 256, 286sh, 371 (NaOMe) 286, 432 (AlCl_3_)272, 302sh, 451 (AlCl_3_ /HCl) 268, 299sh, 356 (NaOAc) 255, 292sh, 387 (NaOAc-H_3_ BO_3_) 267, 389.

**Compound 8**

Yellow microcrystalline powder.

mp: 317-319°C.

Rf: 0.5 (chloroform-methanol 9:1).

IR (KBr): 3300, 3050, 2920, 1600, 1640, 1510, 1360, 1295, 1060, 910.

UV-Vis λ_max_ nm: (MeOH) 260, 368 (NaOMe) 272, 326sh, 406 (AlCl_3_)272, 446 (AlCl_3_ /HCl) 266, 430 (NaOAc) 272, 404 (NaOAc-H_3_ BO_3_) 260, 384.

**Compound 9**

Yellow microcrystalline powder.

mp: 230-232°C.

Rf: 0.6 (chloroform-methanol 8:2).

IR (KBr): 3300, 3050, 2920, 1600, 1640, 1510, 1360,1295, 1060, 910.

UV-Vis λ_max_ nm: (MeOH) 268, 267sh, 344 (NaOMe) 262, 404 (AlCl_3_)276, 330sh, 354sh, 384 (AlCl_3_ -HCl) 274, 300sh, 352sh, 382 (NaOAc) 268, 352sh 406 (NaOAc-H_3_ BO_3_) 264, 338.

**Compound 10**

Yellowish-white amorphous powder.

Rf: 0.23 [ethyl acetate-methanol-water-formic acid (100:16:12:1:0.1 v/v/v/v)].

IR (KBr): 2800, 1620, 1420, 1300, 1310, 1200, 1180, 1150.

UV-Vis λ_max_ nm: (MeOH) 290, 326

^1^H-NMR (300 MHz, DMSO): 1.59 (1H, dd, *J* = 15 and 4 Hz, H-6 ax), 1.78 (2H, m, H-2 ax and eq), 1.94 (1H, dd, *J* = 13 and 9 Hz, H-6 eq), 3.79 (1H, br s, H-4), 3.94 (1H, br s, H-5), 5.14 (1H, m, H-3), 6.18 (1H, d, *J* = 15.9 Hz, H-8’), 6.73 (1H, d, *J* = 6.6 Hz, H-5’), 6.94 (1H, dd, *J* = 8.1 and 2 Hz, H-6’), 7.04 (1H, br s, H-2’), 7.40 (1H, d, *J* = 15.9 Hz, H-7’).

**Compound 11**

Yellow amorphous powder.

mp: 190-192°C.

Rf: 0.3 [ethyl acetate-methanol-water-formic acid (100:16:12:1:0.1 v/v/v/v)].

IR (KBr): 3300, 3050, 2920, 1600, 1640, 1510, 1360, 1295, 1060, 910.

UV-Vis λ_max_ nm: (MeOH) 258, 300sh, 358. (NaOMe) 268, 328sh, 410 (AlCl_3_)270, 306sh, 426 (AlCl_3_ /HCl) 268, 298sh, 366, 400 (NaOAc) 264, 300sh, 382 (NaOAc-H_3_ BO_3_) 262, 308sh, 378.

**Compound 12**

Yellow amorphous powder.

Rf: 0.34 [ethyl acetate-methanol-water-formic acid (100:16:12:1:0.1 v/v/v/v)].

IR (KBr): 3300, 3050, 2920, 1600, 1640, 1510, 1360, 1295, 1060, 910.

UV-Vis λ_max_ nm: (MeOH) 256, 270sh, 358. (NaOMe) 273, 328, 415 (AlCl_3_)270, 299sh, 407 (AlCl_3_ /HCl) 267, 298sh, 360 (NaOAc) 274, 316, 387 (NaOAc-H_3_ BO_3_) 257, 390.

^1^H-NMR (300 MHz, DMSO): 0.97 (3H, d, *J* = 5.1 Hz, CH_3_ -6’’’), 3.84 (3H, s, OCH_3_), 4.39 (1H, d, *J* = 2.1 Hz, H-1’’’), 5.42 (1H, d, *J* = 7.2 Hz, H-1’’), 6.18 (1H, d, *J* = 2.4 Hz, H-6), 6.37 (1H, d, *J* = 2.1 Hz, H-8), 6.90 (1H, d, *J* = 8.7 Hz, H-5’), 7.50 (1H, dd, *J* = 1.2, 6.6 Hz, H-6’), 7.53 (1H, s, H-2’).

^13^C-NMR (75 MHz, DMSO-*d*_6_): 66.84 (C-6’’), 70.09 (C-4’’),74.27 (C-2’’),75.91 (C-5’’), 76.39 (C-3’’), 101.22 (C-1’’), 17.68 (C-6’’), 68.26 (C-5’’’) 70.29 (C-2’’’), 70.58 (C-3’’’), 71.79 (C-4’’’), 100.86 (C-1’’’), 93.60 (C-8), 98.78 (C-6), 103.88 (C-10), 115.07 (C-2’), 116.22 (C-5’), 121.03 (C-6’), 122.26 (C-1’), 133.00 (C-3), 144.73 (C-3’), 146.87 (C-2), 148.43 (C-4’), 156.37 (C-9), 161.14 (C-5), 164.44 (C-7), 178.07 (C-4).

**Compound 13**

Yellow amorphous powder.

Rf: 0.5 [ethyl acetate-methanol-water-formic acid (100:16:12:1:0.1 v/v/v/v)].

IR (KBr): 3300, 3050, 2920, 1602, 1640, 1510, 1360, 1295, 1064, 915.

UV-Vis λ_max_ nm: (MeOH) 269, 302sh, 327, (NaOMe) 276, 295sh, 364 (AlCl_3_)259sh, 277, 292sh, 380 (AlCl_3_ /HCl) 260sh, 279, 296sh, 352, 384 (NaOAc) 278, 297sh, 358 (NaOAc-H_3_ BO_3_)267, 309sh, 331.

^1^H-NMR (300 MHz, DMSO): 3.70 (3H, s, OCH_3_), 3.74 (3H, s, OCH_3_), 4.66 (1H, d, *J* = 9.6 Hz, H-1’’), 6.75 (1H, s, H-3), 6.89 (2H, d, *J* = 8.2 Hz, H-3’,5’), 7.98 (2H, d, *J* = 8.2 Hz, H-2’, 6’).

**Figure 1:**
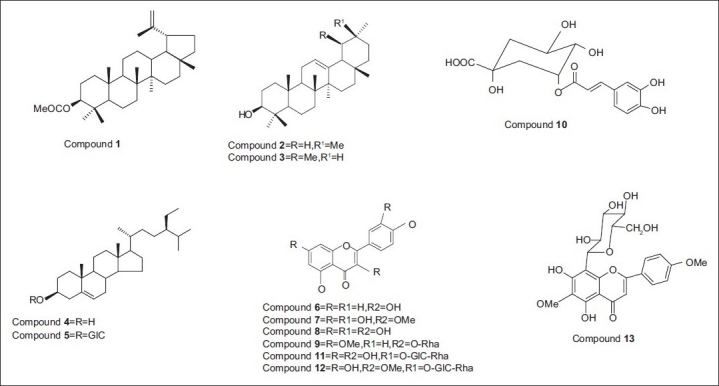
Structures of the isolated compounds

^13^C-NMR (75 MHz, DMSO-*d*_6_): 61.96 (C-6’’), 70.85(C-4’’), 70.86 (C-2’’), 73.45 (C-1’’), 78.83 (C-3’’), 82.01 (C-5’’), 56.61 (4’-OCH_3_), 61.7 (6-OCH_3_), 102.41 (C-3), 103.96 (C-8), 104.60 (C-10), 116.14 (C-3’,5’), 121.95 (C-1’), 129.36 (C-2’,6’), 133.07 (C-6), 156.05 (C-9), 160.43 (C-5,4’), 162.88 (C-2), 164.11 (C-7), 181.99 (C-4). Structures of the isolated compounds are shown in Figure 1.

### Chemicals

Insulin (Humulin^®^), regular, soluble human insulin injection, Lilly Company, USA.

Diamicron^®^ tablets, Servier Egypt Industries Limited, Egypt.

**Streptozitocin and doxorubicin,** Sigma Company, USA.

### Measurements of cytotoxicity by sulfrhodamine B assay

Cytotoxicity was tested using the method of Skehan *et al*.[2] on 2 human cell lines, colon cancer cell line (HCT116), and breast cancer cell line (MCF-7). The IC_50_ (dose of the extract, which reduces survival to 50%) and IC_10_ (dose of the extract, which reduces survival to 10%) for each tested sample were calculated and recorded in Table 1 and compared with the standard drug doxorubicin.

**Table 1:** *In vitro* cytotoxicity of *Moringa peregrina* (Forssk.) Fiori

Compounds	IC_50_ (μg)	
	MCF-7	HCT116
*n*-Hexane fraction	0.40	1.01
Chloroform fraction	0.81	0.54
Ethyl acetate fraction	0.54	1.01
*n*-Butanol fraction	0.54	0.47
Compound 1	1.04	2.89
Compound 2	2.48	2.05
Compound 3	2.35	2.82
Compound 4	2.9	3.05
Compound 5	8.52	3.22
Compound 6	2.01	2.65
Compound 7	1.50	1.90
Compound 8	1.48	1.86
Compound 10	2.21	3.89
Compound 12	1.80	3.70
Compound 13	2.62	3.00
Doxorubicin	0.70	1.17

IC_50_: the concentration that caused 50% death of the cancer cells. MCF-7, breast cancer cell line; HCT116, colon cancer cell line

### Determination of LD_50_

The LD_50_ of the aqueous and ethanolic extracts was calculated according to Karber (1931).[3]

### Antihyperglycemic activity

#### Animals grouping and administration of extracts

Healthy albino Wistar rats (males and females) obtained from the animal house were housed throughout the experiment in polycarbonated cages and the housing facility was maintained at standard conditions: temperature (28°C ± 2°C), relative humidity (50% ± 5%), and a 12:12 h light:dark cycle. Water and commercial palletized diet were available to the animals *ad libitum* throughout the treatment period. The rats were allowed 1 week to acclimatize to pharmacology departmental animal house.

### Induction of diabetes

#### Streptozitocin-induction of diabetes

Rats were rendered diabetic by injecting a freshly prepared streptozotocin (60 mg/kg, i.p.; dissolved in 0.1 M acetate buffer; pH 4.5).[4]

#### Experimental design for antihyperglycemic activity

Antihyperglycemic activity of *M. peregrina* ethanolic (E) and aqueous (A) extracts were studied after oral administration of 25 mg/kg body weight (b.wt.) in streptozitocin diabetic rats, and compared with the standard group taking 1.44 mg/kg b.wt. of Diamicron^®^ tablets orally. And, the antihyperglycemic activity of the n-hexane (HF), chloroform (CF), ethyl acetate (CF), and *n*-butanol (BF) fractions of the ethanolic extract were tested through intraperitoneal injection of a dose of 50 mg/kg b.wt. and compared with a standard group taking 1 U/kg b.wt. of insulin subcutaneously in rats. Blood samples were collected before treatment and 0.5, 1, 2, and 3 h after material administration. The collected blood samples were centrifuged at 2000 *g* for 5 min for serum separation. The samples were analyzed for serum glucose content by using glucose-oxidase/peroxidase method[5,6] with optical density measured at 505 nm using a visible spectrophotometer, and the results are recorded in Tables 2 and 3.

**Table 2:** Antihyperglycemic effect of the aqueous and ethanolic extracts of *M. peregrina* (Forssk.) Fiori

Group	Zero	30 min	1 h	2 h	3 h
Nontreated	447 ± 8.2	449 ± 9.3^c^	464 ± 6.8^c^	470 ± 4.6^c^	482.8 ± 6.1^c^
Treated with aqueous extract	434 ± 17.1	374 ± 2.5	240 ± 5.5^a^	194 ± 12.1^a^	131.6 ± 6.7^a^
Treated with ethanolic extract	417 ± 34.9	404 ± 49.9	346.8 ± 62.9	233.4 ± 28.1^a^	121.2 ± 22.2^a^
Treated with Diamicron^®^	436 ± 9.3	212 ± 3.7^a^	134 ± 2.4^a^	124 ± 2.4^a^	118 ± 2^a^

a Significantly different from nontreated value at *P* < 0.001

c Significantly different from Diamicron^®^ value at *P* < 0.001

**Table 3:** Effect of intraperitoneal injection of different fractions of ethanolic extracts of the aerial parts of *Moringa peregrina* (Forssk.) Fiori

Group	Zero	30 min	1 h	2 h	3 h
Nontreated	447 ± 8.2	449 ± 9.3^b^	464 ± 6.8^b^	470 ± 4.6^b^	482.8 ± 6.1^b^
Treated with HF	393.7 ± 63.1	88.92 ± 14.1^a^	92.8 ± 14.7^a^	120 ± 19.1^a^	120.45 ± 18.8^a^
Treated with CF	423 ± 63.1	436 ± 57.9^b^	374.5 ± 61.5^b^	382.6 ± 61.2^b^	383.4 ± 62.5^b^
Treated with EF	399 ± 64.2	361.1 ± 57.4^b^	355.2 ± 53.6^b^	352.49 ± 56.7^b^	368.6 ± 58.7^b^
Treated with BF	432 ± 33.7	410 ± 31.5^b^	411.8 ± 31.4^b^	414.2 ± 31.5^b^	433.8 ± 31.4^b^
Treated with insulin	450.3 ± 23	253.2 ± 15.8^a^	143 ± 6.4^a^	62.2 ± 2.6^a^	68.6 ± 3^a^

HF, n-hexane; CF, chloroform; EF, ethyl acetate; and BF, *n*-butanol

a Significantly different from nontreated value at *P* < 0.001

b Significantly different from insulin value at *P* < 0.001

### Statistical analysis

All data are expressed as mean ± SE and the statistical significance was evaluated by one-way analysis of variance (ANOVA).[7] The values are considered significantly different when *P* values were less than 0.01.

## RESULTS AND DISCUSSION

Thirteen compounds were obtained from the different fractions of the ethanolic extract of the dried aerial parts. Five compounds were isolated from the n-hexane fraction, compounds 1-4 were identified as lupeol acetate (1), β-amyrin (2), α-amyrin (3), and β-sitosterol (4) from their IR, mass spectra,[8–10] and by direct comparison of mps and co-chromatography with authentic samples. Compound 5 was identified as β-sitosterol-3-O-β-d-glucoside where the data were concordant with that reported in the literature.[9–11] Four compounds were isolated from the ethyl acetate fraction, which were identified as apigenin (6), rhamnetin (7), quercetin (8), and chryseriol-7-O-rhamnoside (9); the compounds were identified from the IR, UV data, and by direct comparison with authentic samples (co-TLC).

Four compounds (10, 11, 12, and 13) were isolated from the *n*-butanol fraction, compound 10 was identified as 3-caffoeylquinic acid [neo-chlorogenic acid] by comparing its IR, UV spectral data,^1^H-NMR spectrum, and^1^H-^1^H COSY with the published data.[12–14] Compound **12** was identified as rhamnetin-3-O-rhamnoglucoside through its IR, UV spectral data,^1^H-NMR, and^13^C-NMR spectra.[14,15] Compound **11** was identified as quercetin-3-O-rutinoside from its IR, UV data, and by direct comparison with an authentic sample (co-TLC).

The IR, UV spectral data, and^1^H and^13^C-NMR spectrum of compound **13** indicated the presence of an acacetin nucleus[15–17] with the absence of H-6 and H-8, in addition to a methoxy group and an anomeric proton. Two doublets at δ 6.89 and 7.98 ppm each was integrated as two protons and assigned to H-3’, H-5’ and H-2’, H-6’, respectively. A singlet at δ 6.75 ppm was integrated as 1 proton and assigned to H-3. Finally, a singlet at δ 3.74 ppm was integrated as 3 protons and assigned to the methoxy group at 4’. The appearance of the anomeric proton at δ 4.66 ppm with a large coupling constant 9.6 Hz and the absence of H-8 in the^1^H-NMR spectrum indicated a β-linked sugar and glycosylation was at C-8.[17] This was confirmed from the^13^C-NMR spectrum through the upfield shift of the anomeric carbon C-1’’, which appeared at δ 78.83 ppm and the downfield shift of C-8 (by about 9 ppm), which appeared at δ 102.41 ppm.[15] The absence of H-6 and the presence of a singlet at 3.70 integrated as 3 protons assigned to a methoxy group, which appeared in^13^C-NMR spectrum at δ 61.7 ppm suggested the presence of a methoxy group at C-6. This was confirmed through the downfield shift of C-6 (by about 35 ppm), which appeared at 133.07 ppm.[15] From the above data, compound 13 may be 6-methoxy-acacetin-8-C-β-glucoside.

On measuring the acute toxicity, the plant showed no toxic symptoms and no deaths after oral and intraperitoneal administration of the tested doses. The different fractions of the ethanolic extracts of the aerial parts of *M. peregrina* had a potent cytotoxic activity against the 2 tested human cell lines Colon cancer cell line (HCT116) and breast cancer cell line (MCF-7) [Table 1]. This is obvious from the small IC_50_ of the different fractions, which were comparable to that of doxorubicin. The major isolated compounds were also tested for their cytotoxic activity, and they showed a potent activity against the 2 cancer cell lines.

On the other hand, the aqueous (A) and ethanolic (E) extracts of the aerial parts of *M. peregrina* significantly decreased blood glucose level of streptozitocin diabetic rats [Table 2] and their effects were comparable to the oral antidiabetic reference drug Diamicron^®^. Accordingly, the n-hexane (HF), chloroform (CF), ethyl acetate (CF) and *n*-butanol (BF) fractions of the ethanolic extract were also tested for their antihyperglycemic effect through intraperitoneal injection of a dose of 50 mg/kg b.wt. using insulin as a standard antidiabetic [Table 3]. The n-hexane fraction (HF) was the only fraction that showed a highly significant antihyperglycemic activity. Its effect started 30 min after injection and decreased the blood glucose level by 64%-77.44%. This effect remains significant after 3 h from injection. This effect could be attributed to the lupeol acetate and β-sitosterol, which were isolated from the n-hexane fraction and were reported to possess antihyperglycemic effect.[18]
